# A case report of a giant rectal adenoma causing secretory diarrhea and acute renal failure: McKittrick-Wheelock syndrome

**DOI:** 10.1186/s12893-016-0153-2

**Published:** 2016-06-07

**Authors:** Annamaria Agnes, Domenico Novelli, Giovanni Battista Doglietto, Valerio Papa

**Affiliations:** Division of Surgery, Miulli General Hospital, S.P. 127 Acquaviva-Santeramo km 4, 70021 Acquaviva delle Fonti, Italy; Catholic University, School of Medicine, Largo Agostino Gemelli n.8, 00168 Rome, Italy; Division of Digestive Surgery, Gemelli Hospital, Catholic University, School of Medicine, Largo Agostino Gemelli n.8, 00168 Rome, Italy

**Keywords:** Villous adenoma, Secretory polyp, Chronic diarrhea, Acute renal failure, Electrolyte depletion

## Abstract

**Background:**

The McKittrick-Wheelock syndrome is a rare depletion syndrome caused by a secretory villous adenoma or a carcinoma of the rectosigmoid tract. An aggressive hydroelectrolyte rebalancing is often needed, and curative treatment is obtained only with complete removal of the lesion, by endoscopy or surgery. Low clinical suspicion often delays the diagnosis, resulting in detrimental complications.

**Case presentation:**

We report the case of a 75-year-old woman, presenting to the emergency department with acute renal failure and electrolyte imbalance, reporting an history of recurrent episodes of dehydration and chronic diarrhea. After being admitted to the nephrology department she underwent diagnostic investigation that revealed the presence of a giant adenoma of the rectum. The patients received supportive therapy and was subsequently treated with surgery, with a favorable outcome.

**Conclusions:**

A prompt diagnosis plays an important role in the treatment of McKittrick-Wheelock syndrome. We describe a case of this condition in detail and review the related literature, underlining the typical diagnostic features and exploring the possible therapeutic options.

## Background

Adenomatous polyps of the sigmoid and rectum are usually characterized by a lack of symptoms, although sometimes they present with tenesmus and rectal bleeding [1]. In rare cases, these tumors cause a clinical picture characterized by chronic diarrhea, severe dehydration and electrolyte depletion, which may lead to several systemic complications, namely McKittrick-Wheelock syndrome [2]. We describe the case of a 75-year-old woman admitted to our hospital with a clinical history that raised the suspect of this condition. She underwent examinations that confirmed the diagnosis, and she was successfully treated with surgery. We present this case to underline the necessity of maintaining an high clinical suspicion for this syndrome in patients with an history of unexplained chronic diarrhea, especially given that the condition has a good prognosis when diagnosed and treated in a timely manner.

## Case presentation

A 75-year-old woman was admitted to the emergency department with vomit, diarrhea, confusion and epigastric pain. She had a diagnosis of chronic renal insufficiency for several months, and she recently suffered several episodes of urinary tract infection and acute renal failure due to severe dehydration consequent to diarrhea. Furthermore, she reported consistent weight loss in the last period. She had hypertension, cardiac insufficiency, chronic pulmonary obstructive disease, hiatal hernia and an history of cardiac arrhythmias. On admission, blood pressure was 140/85 mmHg and pulse rate was 95. On physical examination, abdomen was nontender. Abdominal ultrasound was negative. An electrocardiograph was normal. Laboratory analysis were significant for hyponatriemia (124 mmol/L, normal range: 135–145 mmol/L), hypokalemia (3.1 mmol/L, normal range: 3.5–5.0 mmol/L), high creatininemia (318.24 μmol/L, normal range: 53–106 μmol/l), hyperazotemia (188 mg/dl, normal range: 10–50 mg/dl), leukocytosis (10.810 x 10^9/L, normal range 4.0–10.0 x 10^9/L), and proteinuria (2.5 g/day, normal value: <0.15 g/day). After adequate rehydration and PPI infusion epigastric pain subsided. She was admitted to the nephrology department, where she was treated with intravenous 0.9 % saline and potassium infusion. A complete normalization of her biochemistry, followed by normalization of her creatinine values, occurred only after 10 days of intravenous therapy. In light of her persistent, mucous diarrhea a digital examination was performed, documenting a circumferential lesion of the rectum, with mucosal involvement of the anal canal up to 1 cm from the anal verge, and a colonoscopy was ordered.

**Fig. 1:**
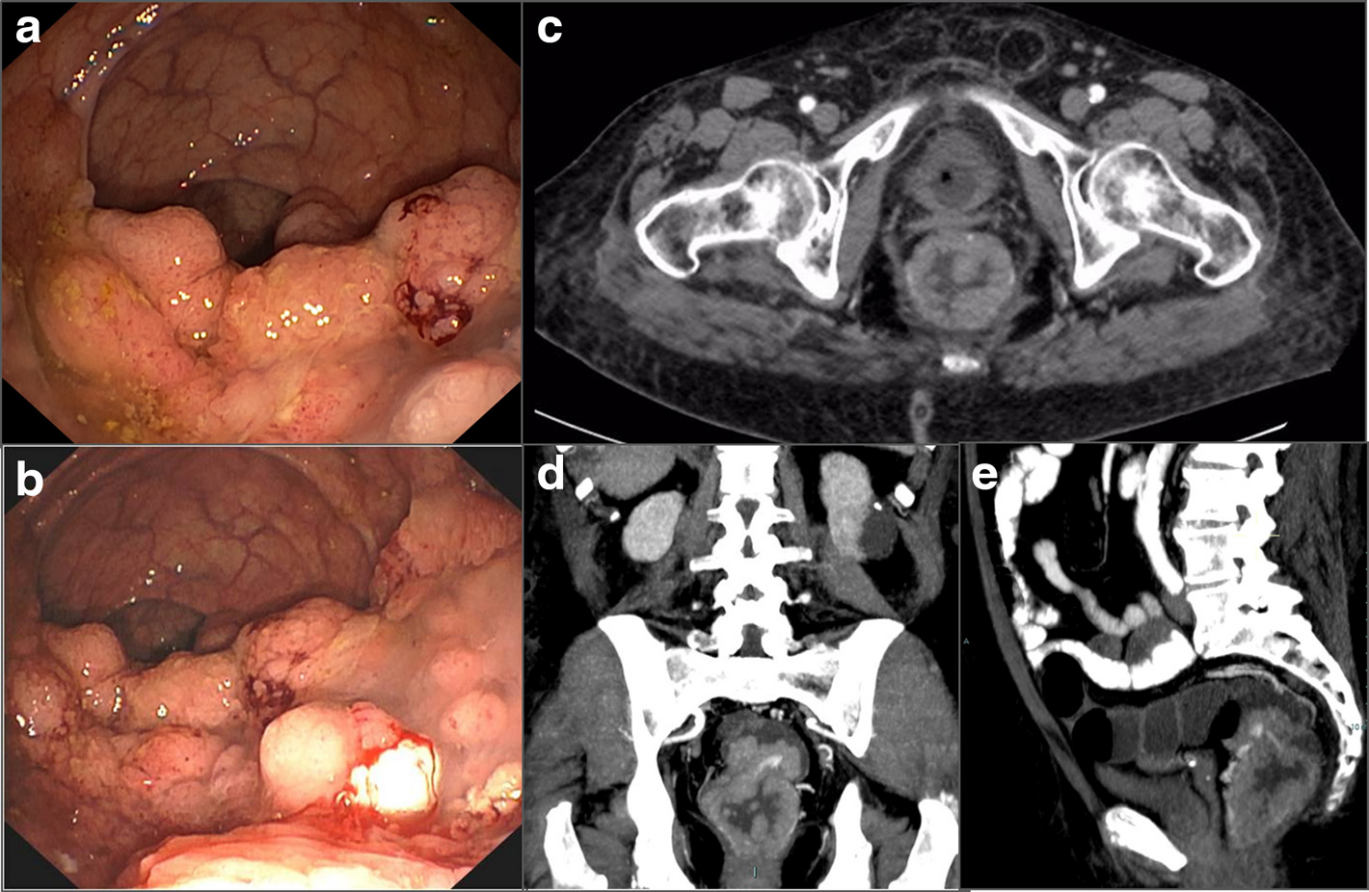
Diagnostic features of the giant rectal polyp. Panel **a**-**b** Colonoscopy revealing a large endoluminal polypoid mass involving the rectum; Panel **c** abdominal computed tomography revealing the presence of a giant villous tumor occupying the rectal lumen, transversal/axial view; Panel **d** coronal view; Panel **e** sagittal view

The colonoscopy showed sigmoid diverticula and a giant polyp of the rectum, occupying three quarters of the luminal circumference, with a cranial-caudal extension of 8–9 cm and (Fig. 1). Considering the typical clinical presentation, McKittrick-Wheelock syndrome was diagnosed.

A biopsy was performed on the polyp, and pathology showed a villous adenoma with focal high-grade dysplasia. Subsequently, the patient underwent a contrast-enhanced CT scan of the thorax and abdomen for staging of the disease. The CT scan did not show any signs of mural involvement, nor sphincterial or mesorectal involvement or signs of distant metastases (Fig. 1). She was finally transferred to our department and referred to surgery.

**Fig. 2:**
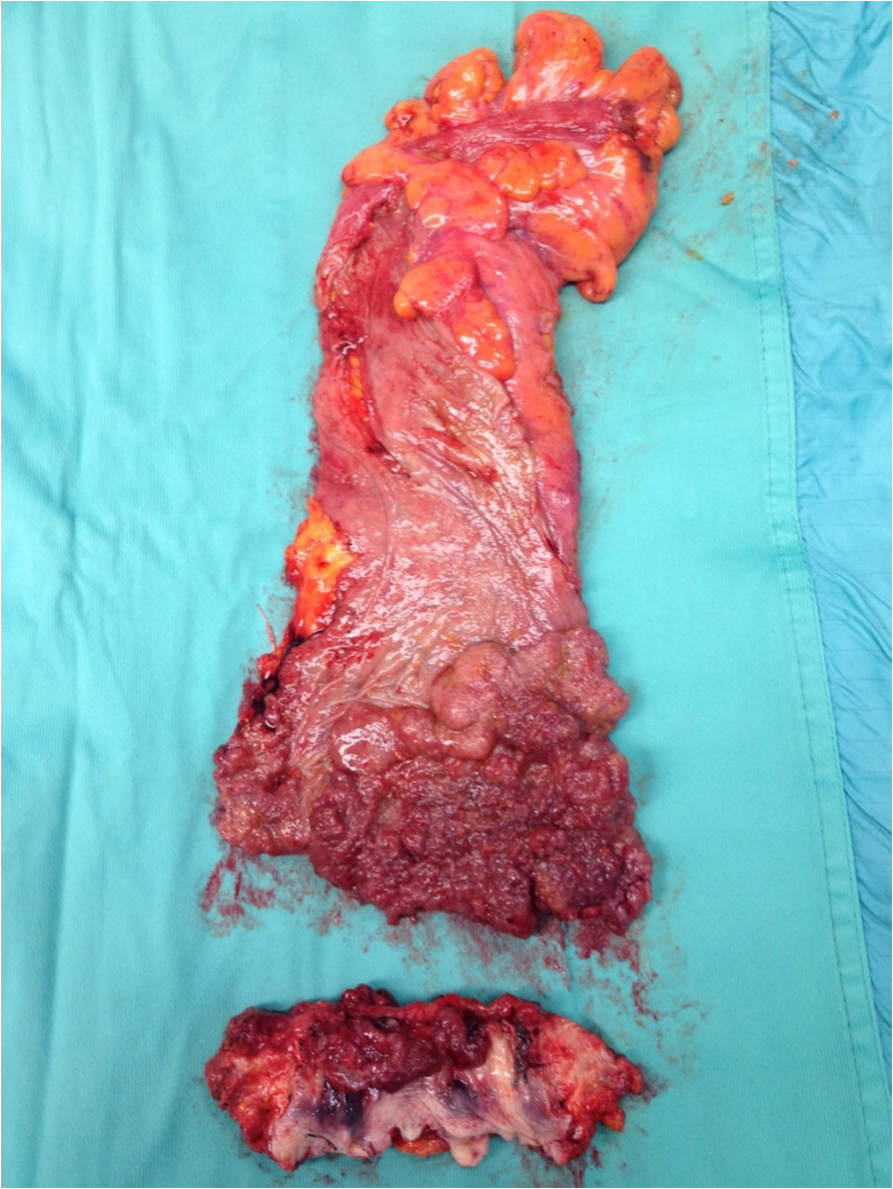
Surgical specimen showing the adenomatous degeneration of the rectal mucosa

Given the dimension and the extension of the polyp transanal surgery was excluded, and considering the good general conditions and continence status of the patient a low anterior rectal resection with mucosectomy of the anal canal had initially been planned. However, an intra-operative frozen section pathological examination showed involvement of the distal margin by intramucosal adenocarcinoma, and a conventional abdomino-perineal resection with end-colostomy was performed (Fig. 2).

Pathological examination on the whole specimen showed a villous adenoma with areas of intramucosal adenocarcinoma and high grade dysplasia (TisN0).

After surgery, the patient showed persistent normalization of physical and serological parameters and full recovery of her renal function. She did not have any medical or surgical postoperative complication. At 10 months follow-up, she was in good general conditions, without signs of local recurrence, with a regular GI function and no signs of renal insufficiency or electrolyte disturbance.

## Discussion

The McKittrick-Wheelock syndrome is a depletion disorder first described in 1954 [2]. The cause is a colorectal tumor, most often a villous adenoma greater than 4 cm [3]. The tumor secretes a conspicuous amount of sodium and potassium, causing a mucous diarrhea that may lead to severe dehydration and refractory electrolyte imbalance (hyponatriemia, hypokaliemia, hypochloremia), with episodes of acute renal failure. Apart from renal insufficiency, symptoms of this syndrome could also be related to electrolyte depletion (vomit, confusion, asthenia, cardiac arrhythmias). Patients with an unrecognized long-term syndrome may even necessitate hemodialysis or be at risk of life-threatening events [2–5].

Villous adenomas causing this syndrome are most often located in the sigmoid colon or in the rectum. They secrete sodium and potassium, and the sodium gradient leads to a secretive diarrhea. The distal location of the tumor does not allow an adequate compensatory reabsorption mechanism, given the absence of normal colonic mucosa below [4, 6–8].

Sodium and water hypersecretion seem due to an abnormal production of Prostaglandin E2 (PGE2) by the villous adenoma cells. PGE2 acts as a secretagogue, inhibiting sodium reabsorption and thus causing secretive diarrhea. Indeed, some authors have proposed pharmacological treatment with non-steroidal antinflammatory agents (NSAIDS), inhibitors of the Cyclooxygenase-2 (COX-2) pathway or somatostatin analogues to aid normalization of the electrolyte imbalance of these patients and to treat individuals who refuse surgery [3, 7, 9, 10]. However, given the anecdotal nature of this evidence, we believe that this kind of pharmacological treatment should be reserved exclusively to McKittrick-Wheelock patients not amenable of surgical resection and extremely cautiously in substitution to hydroelectrolyte rebalancing as a bridge to surgery.

Diagnosis could be difficult to assess immediately, because villous adenomas of the sigmoid and rectum are often characterized by a lack of symptoms and this syndrome is rare, as only 3 % of villous adenomas are hypersecretory [6, 11]. It has been suggested that the triad of prerenal failure, electrolyte disorder and chronic diarrhea should always prompt a complete colonoscopy [4].

The first step in therapy should consist of hydroelectrolyte rebalancing, followed by removal of the lesion causing the syndrome [4]. In the past, some authors have addressed the possibility of selectively using endocavitary irradiation for the treatment of rectal adenomas, but data had shown a local recurrence rate of almost 32 %, [3, 12] so this treatment is hardly advisable. Endoscopy and transanal surgery, when feasible, could be a satisfactory treatment for smaller lesions without signs of malignancy, though a high-risk of cancerization of these lesions has been reported. Indeed, almost 80 % of these villous adenomas have been reported to host foci of adenocarcinoma [1, 4, 10, 11]. Consequently, in the majority of cases, the most appropriate treatment is surgery and should consist of a formal oncologic resection. Complete removal of the lesion prevents progression of the tumoral disease and resolves the depletion syndrome [4, 6, 7]. In addition, some authors have reported successful outcomes obtained with laparoscopic rectal resections and intersphincteric rectal resections [13, 14].

## Conclusions

Our patient had a late diagnosis, as the syndrome was suspected only after some months since her first symptoms. Fortunately, the giant polyp had only initial signs of cancerization and she had not developed an invasive cancer at the time of diagnosis. After removal of the mass her clinical picture reversed in a short time, and her renal insufficiency and cardiac arrhythmias completely resolved. The McKittrick-Wheelock syndrome is an entity surgeons, gastroenterologist and nephrologists should be aware of, as to obtain a prompt diagnosis in typical cases and to intervene with an appropriate therapy, resolving a condition that may develop life-threatening complications and that, if left untreated for a long time, could evolve in invasive cancer.

## Abbreviations

COX-2, Cyclooxygenase-2; PGE2, Prostaglandin E2
